# Selection of Peptides Targeting Helix 31 of Bacterial 16S Ribosomal RNA by Screening M13 Phage-Display Libraries

**DOI:** 10.3390/molecules16021211

**Published:** 2011-01-28

**Authors:** Tek N. Lamichhane, N. Dinuka Abeydeera, Anne-Cécile E. Duc, Philip R. Cunningham, Christine S. Chow

**Affiliations:** 1 Department of Chemistry, Wayne State University, Detroit, MI 48202, USA; 2 Department of Biological Sciences, Wayne State University, Detroit, MI 48202, USA

**Keywords:** Helix 31, modified nucleotides, peptides, phage display, 5-methylcytidine, 2-methylguanosine

## Abstract

Ribosomal RNA is the catalytic portion of ribosomes, and undergoes a variety of conformational changes during translation. Structural changes in ribosomal RNA can be facilitated by the presence of modified nucleotides. Helix 31 of bacterial 16S ribosomal RNA harbors two modified nucleotides, m^2^G966 and m^5^C967, that are highly conserved among bacteria, though the degree and nature of the modifications in this region are different in eukaryotes. Contacts between helix 31 and the P-site tRNA, initiation factors, and ribosomal proteins highlight the importance of this region in translation. In this work, a heptapeptide M13 phage-display library was screened for ligands that target the wild-type, naturally modified bacterial helix 31. Several peptides, including TYLPWPA, CVRPFAL, TLWDLIP, FVRPFPL, ATPLWLK, and DIRTQRE, were found to be prevalent after several rounds of screening. Several of the peptides exhibited moderate affinity (in the high nM to low µM range) to modified helix 31 in biophysical assays, including surface plasmon resonance (SPR), and were also shown to bind 30S ribosomal subunits. These peptides also inhibited protein synthesis in cell-free translation assays.

## 1. Introduction

RNA-protein and RNA-RNA interactions are necessary for the main cellular activities of every living organism [1]. Natural RNA modifications often play a role in modulating or fine-tuning these interactions [2]. For example, lack of modification *N*^6^,*N*^6^-dimethyladenosine at positions 1518 and 1519 of the small subunit ribosomal RNA (rRNA) alters the RNA structure and affects 30S subunit assembly [3,4], and tRNA modifications have been shown to fine-tune the affinity of the various tRNAs for their ribosome binding sites [5]. Although the distribution of modified nucleotides appears to be random throughout the rRNA secondary structure, three-dimensional structures show that they are clustered in the functional center of the ribosome such as the peptidyl-transferase center (PTC), decoding region, aminoacyl- and peptidyl-tRNA binding sites (A and P sites), peptide exit tunnel, and intersubunit bridges [6,7]. Loss of rRNA modifications affects a variety of important processes such as tRNA binding, subunit assembly, and translational fidelity [8,9,10,11,12]. Resistance to antibiotics can also arise if specific modifications are either lacking or inserted [13,14]. These facts highlight the possibility of using modified RNAs as potential drug targets. There is an urgent need for identification of new drug target sites because of the emergence of multiple drug resistance [15,16,17], as well as functional mutations at the existing target sites [18,19,20]. 

**Figure 1:**
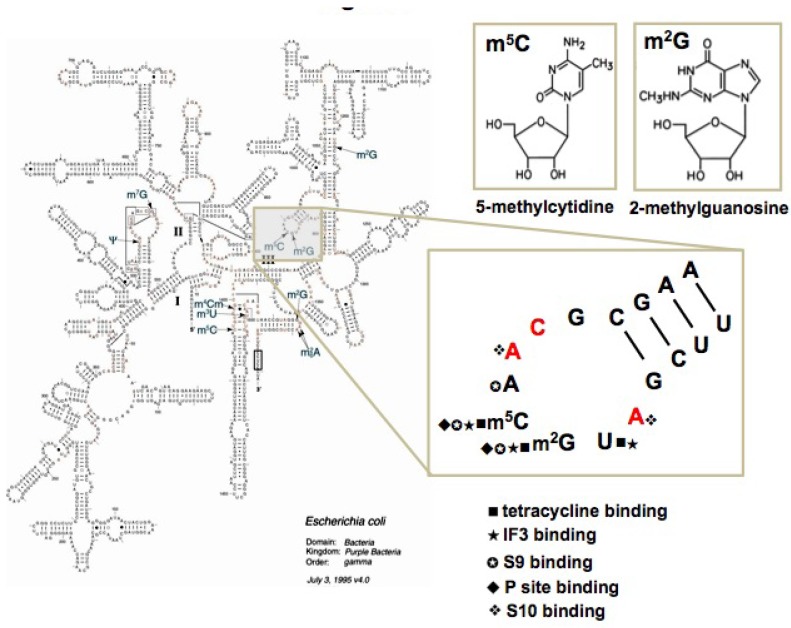
Location of helix 31 on the secondary structure of *E. coli* 16S rRNA [21]. The enlarged helix 31 shows the positions of the modified nucleotides m^2^G966 and m^5^C967, and sites for interactions with drugs, ribosomal proteins, tRNA, and factors. Nucleotides in red are universally conserved.

Helix 31 (h31, or 970 loop) of bacterial 16S rRNA contains two modified nucleotides, 2-methylguanosine at position 966 (m^2^G966) and 5-methylcytidine at residue 967 (m^5^C967) (Figure 1) [21,22,23]. A modified nucleotide is present at position 966 (*E. coli* numbering) in all three phylogenetic domains, but the type of modification is domain specific [22,23]. The corresponding position in the small subunit rRNAs of the *Eukarya* and *Archaea* contains hypermodified nucleotides 1-methyl-3-(3-amino-3-carboxypropyl)pseudouridine (m^1^acp^3^Ψ) and 3-(3-amino-3-carboxypropyl)uridine (acp^3^U), respectively [23,24,25]. The m^5^C modification at position 967 is present only in the *Bacteria* [22,25]. Although disruption of genes responsible for m^2^G966 and m^5^C967 modification does not significantly alter growth rate, it does reduce fitness in competition with wild-type cells [26,27]. Mutational studies of h31 loop nucleotides revealed that changes in loop structure due to disruption of critical stacking interactions strongly inhibit ribosome function *in vivo* [28]. Single mutations at m^2^G966 or m^5^C967, however, produce more protein *in vivo* than wild-type ribosomes. The 966 and 967 single mutants were specifically complemented by over-expression of initiation factor 3 (IF3), suggesting that modification of these residues is important for IF3 binding and for the proper initiation of protein synthesis [28]. In bacteria, the modified nucleotides of h31 are located at the binding site of IF3, tetracycline, and the anticodon-stem loop of the P-site tRNA [29,30]. The presence of these distinct types of modifications in h31 of bacteria and eukaryotes (e.g., methylation vs. pseudouridylation and hypermodification) suggested that this region could potentially be used as a novel target site for drug discovery. 

Peptides that disrupt critical functional interactions by binding to a specific bacterial rRNA motif could be potential drug leads. With the development of new technologies, peptide-based drugs are taking a leading role in drug discovery and are now viable alternatives to antibodies and small molecules as potential drugs [31,32]. Peptides have several advantages over small molecules, such as ease of tissue penetration, high specificity, and low toxicity for certain sequences [33]. Although peptides have some drawbacks such as short half lives, susceptibility to protease degradation, and low transport through the blood-brain barrier [33], the use of peptidomimetic chemistry, in which the functional groups and backbones of the peptides are altered, has been shown to address some of these limitations [34,35].

Peptides that recognize and bind to specific RNA structural motifs can be discovered by a powerful method known as phage display [36]. A high diversity of peptides in the library can be screened against a broad range of biological targets [37]. After the development of phage-display technology in 1985, most of the work that followed involved the isolation of ligands using proteins as targets. In contrast, the use of phage display to identify peptides that interact with RNA targets is relatively new [38,39]. Peptides that mimic specific protein motifs, such as those observed in the well-known Tat-TAR and Rev-RRE systems, have been studied in detail [40,41,42]. Usually the small peptides present in commercial phage-display libraries are unstructured under physiological conditions, but have been shown to adapt to the structures of the target RNA [40,43]. The U1 RNA-binding antibody fragments were isolated from autoimmune human-derived bacteriophage display libraries [44,45]. Similarly, peptide ligands for target RNA structures from the HIV-1 packaging signal were identified and their specificity was characterized [46,47]. Peptides with modest binding affinity to the A site of 16S rRNA were isolated, and their binding site was shown to be overlapping with that of the aminoglycoside antibiotic paramomycin, indicating that peptides can bind to a validated drug target site [48]. Peptides have also been selected to bind to the 530 loop of 16S rRNA [49]. Peptides targeting modified nucleotides in tRNA was demonstrated by Agris and coworkers [50,51,52,53], but to our knowledge, no work has been done to target specifically the modified nucleotides of ribosomal RNA.

The aim of this research was to identify peptide ligands that bind specifically to wild-type h31 of bacterial 16S rRNA. Peptides were selected after several rounds of screening with a commercial phage library and characterized through biophysical and biochemical studies. These results illustrate the potential use of peptides as drug leads to target modified nucleotides in ribosomal RNA. 

## 2. Results and Discussion

### 2.1. Target preparation

**Figure 2:**
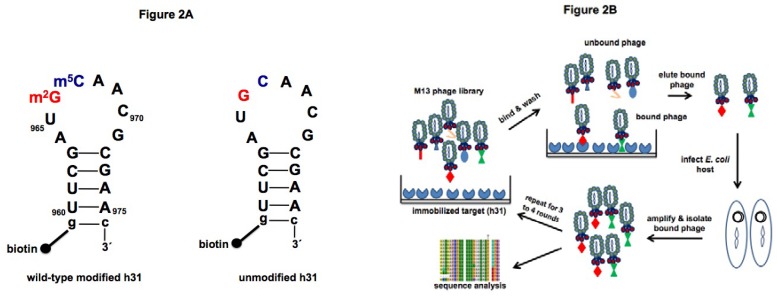
A) RNA targets for phage display. The 5'-biotinylated, wild-type (modified) h31 (left) and unmodified h31 (right) constructs are shown. The sequences are derived from the *E. coli* 16S rRNA, positions U960–A975. Positions 966 and 967 are highlighted in red and blue, respectively. An extra closing base pair (g-c) was added at the end of the stem to increase hairpin stability. B) Schematic diagram of the phage display screen. The cycle starts with exposure of the phage-display peptide library to the target RNA molecules, which are immobilized on a streptavidin surface. Washing removes unbound phage. Bound phage are eluted by denaturation, and amplified in *E. coli*. Cycles are repeated until target-specific peptides are isolated.

Two h31 constructs were used as bait for peptide screening in phage display (Figure 2A). The 18-nucleotide (nt) RNA construct representing wild-type h31 with modified nucleotides m^2^G and m^5^C and the corresponding unmodified RNA were synthesized chemically as described previously [54]. The 5' ends of the RNA oligonucleotides were biotinylated, allowing them to be immobilized onto streptavidin-coated beads. Free (without biotin) unmodified h31 was also used for counter selection in later rounds during the screening step.

### 2.2. Screening

Duplicate samples of wild-type h31 (30 to 50 pmoles) on magnetic beads and one control (beads only) without RNA were employed for selection. A similar selection was carried out with the corresponding unmodified h31. To identify peptides that bind to h31 (either modified or unmodified), a random heptapeptide library (Ph.D.-7^TM ^from New England Biolabs, 10 µL, 2 × 10^13^ pfu/mL) was added to the biotinylated RNA target affixed to streptavidin-coated beads and the bead mixture was washed to remove unbound and weakly bound phage (Figure 2B). Bound phage were then eluted and amplified for further rounds of screening. After the third and fourth rounds of selection, individual clones were sequenced to identify peptides with affinity for the target. The number of washes and concentration of detergent (Tween-20) were increased (0.1 to 0.5%) in each round of selection to enhance removal of weakly bound and non-specific peptides (Table 1). Free competitor RNA (30 pmoles tRNA) was added in each round to remove peptides that exhibit non-specific RNA binding. A separate counter selection was also carried out in the third round of selection against wild-type h31 in which free unmodified h31 (30 pmoles) was added to the phage and modified h31-streptavidin mixture. The purpose of this step was to enrich for peptides with a preference for the wild-type modified h31 RNA. For each selection, the ratio of bound phage to unbound phage increased until the third round, suggestive of successful enrichment of target-specific peptides (Supplementary Material, Table S1). 

**Table 1:** Biopanning conditions for h31.

Round no.	Round 1	Round 2	Round 3	Round 4
No. of washes (200 µL/wash)	12	18	24	24
Tween-20	0.1%	0.1%	0.3%	0.5%
Target RNA (h31)	50 pmoles	50 pmoles	30 pmoles	30 pmoles
Competitor tRNA	30 pmoles	30 pmoles	30 pmoles	30 pmoles

A positive control experiment to identify peptides that bind to streptavidin was performed by screening the library against streptavidin-coated beads without target RNA. Peptides having the streptavidin-binding motif, HPQ, [55], comprised 65% of the total peptides in the third round, and increased to 85% in the fourth round (Supplementary Material, Table S2). Peptide sequences obtained from the target (modified h31) did not match any of the peptide sequences or motifs isolated from the control beads. 

For each sample of h31, 100 isolates were sequenced. An additional 100 isolates were obtained from the counter selection. Altogether, 300 clones against wild-type h31 were sequenced. The sequences were analyzed by RELIC software [56], which is a bioinformatics server for combinatorial peptide analysis. The predominant sequences obtained from selection against wild-type modified h31 were TLWDLIP, TYLPWPA, ATPLWLK, DIRTQRE, FVRPFPL, and FVRPFAL (Table 2). An identical peptide, TLWDLIP, and a closely related peptide, CVRPFAL, were obtained from the selection in which unmodified h31 was used in a counter selection step in round 3 (Table 3). Sequence analysis revealed peptides containing the -TLW- and -VRP- motifs in both screens using wild-type modified h31, the first without counter selection, and the second including counter selection with unmodified h31. The peptides from the counter selection were anticipated to display specific binding to modified h31 and interact with residues m^2^G966 and/or m^5^C967. Out of the five major peptides selected for further study, three of them (DIRTQRE, CVRPFAL, FVRPFAL) contain arginine at the third position of the seven-amino-acid peptide. The presence of R was thought to potentially enhance interactions with the phosphodiester backbone. Two other peptides that were identified (TLWDLIP and TYLPWPA) contain aromatic tryptophan and tyrosine residues, which have the potential to intercalate with the nucleobases of h31.

**Table 2:** Peptides obtained using modified h31 RNA as a target^a^.

Peptides							Frequency
F	V	R	P	F	P	L	6
F	V	R	P	F	A	L	4
F	V	R	P	Y	A	P	2
F	P	R	T	I	A	P	1
D	I	R	T	Q	R	E	4
D	I	R	T	Q	T	R	2
D	I	R	A	T	Q	A	2
A	T	P	L	W	L	K	5
A	T	P	T	Q	R	E	3
A	T	P	L	Y	L	R	2
T	L	W	D	L	I	P	5
T	L	W	S	F	M	P	3
T	L	W	V	P	S	R	2
T	L	T	T	L	T	N	2
T	L	T	F	F	H	R	2
T	Y	L	P	W	P	A	5
T	Y	L	P	W	P	P	2
T	Y	L	R	A	R	L	2
T	Y	P	F	A	P	W	2
T	Y	L	R	A	R	L	1

^a^Two hundred phage isolates were sequenced after the third round of selection against wild-type modified h31 on streptavidin-coated beads. Only peptide sequences with repeated sequences or common motifs are shown.

**Table 3:** Peptides obtained using modified h31 as a target and unmodified h31 in counter selection in round 3^a^.

Peptides							Frequency
C	V	R	P	F	A	L	5
C	V	R	A	P	T	L	2
T	V	R	P	F	T	L	2
T	L	W	D	L	I	P	2
T	L	W	P	L	S	P	2

^a^ One hundred phage isolates were sequenced after the third round of selection. Only peptide sequences with repeated sequences or common motifs are shown.

Phage selection against unmodified h31 under similar selection conditions revealed two major peptides, HHHPPLA and KPFHNST, after four rounds (Supplementary Material, Table S3). Both peptides are rich in polar residues. None of the peptides isolated from the unmodified h31 target selection matched those from the wild-type modified h31 screen. A small number had a similar three amino-acid motif such as "KPF" and "TPL", but their statistical significance was low. Such a difference in the selected peptides might arise due to alternate RNA loop structures for modified and unmodified h31 or to direct interactions with the modified residues. The peptides from each selection have the potential for specificity to either modified or unmodified h31. 

Previous biophysical studies revealed that the modified nucleotides of *E. coli* h31 slightly destabilize the hairpin [54]. A high-resolution crystal structure of *T. thermophilus* ribosomes in the presence of tRNA and mRNA shows a flipped out conformation of m^2^_2_G966 [57]. Dynamic behavior of the h31 loop could play a role in accommodation of the P-site tRNA, as revealed by the *E. coli* 70S ribosome structure complexed with tRNA and mRNA [58,59]. These observations, along with the fact that different peptide sequences were selected for the modified and unmodified h31 RNAs, suggest that it will be possible to obtain ligands that have specificity for modified rRNAs, and that they may also be able to regulate rRNA conformational changes. 

The fact that convergence to a single sequence or motif in the selection process, as is often observed with protein targets [60,61], was not observed in the case of h31 (for either unmodified or modified RNA) is not surprising given the fact that both the RNA target and the peptides are flexible and can exist in multiple conformational states. Further rounds of selection might have lead to convergence, but were not attempted because we believed that increasing the stringency might lead to peptide sequences that only target the more rigid, common features between modified and unmodified h31, such as the duplex (stem) region, rather than the loop region.

Sequence alignment of the selected peptides with known *E. coli* proteins revealed homology with several RNA modifying enzymes and tRNA synthetases (Supplementary Material, Table S4). All but one of the homologs are RNA-binding proteins, two are known rRNA methyltransferases and one is a predicted methyltransferase. Among our peptide isolates, it is unlikely that selectivity for the modified form of h31 is due to binding of the peptides to the methylated residues only. Instead, to achieve sufficient affinity to survive competition during the screening process, separate portions of each peptide probably interact with both unmodified and modified residues in modified h31. The fact that all but one of the homologs are either RNA-binding proteins, methyltransferases, or both RNA-binding proteins and methylases is consistent with this hypothesis.

### 2.3. Characterization of peptides with a coupled in vitro transcription-translation assay

**Figure 3:**
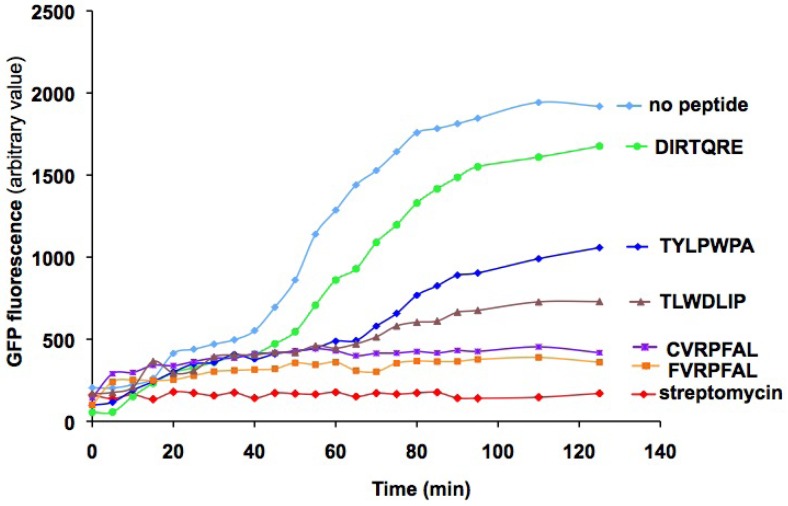
Levels of translation inhibition of green fluorescent protein (GFP) in the presence of selected peptides (1 mM). A DNA template for GFP (plasmid pRSETEmGFP) was employed at a concentration of 10 µg/mL in 15 µL. The production of GFP was monitored over a time interval of 0 to 140 min by relative fluorescence intensity (excitation at 487 nm, emission at 509 nm). The peptides are indicated by colored symbols: no peptide (light blue diamonds), DIRTQRE (green circles), TYLPWPA (dark blue diamonds), TLWDLIP (violet triangles), CVRPFAL (purple squares), and FVRPFAL (orange squares). The streptomycin concentration in the positive control was 0.3 mM (red diamonds).

A cell-free translation assay was used to test the potential inhibitory effects of the peptides on protein synthesis. Different concentrations of peptides were added to *E. coli* S30 extracts and the extent of inhibition was monitored by the measurement of green fluorescent protein (GFP) translation. Streptomycin at 300 µM, which was used as a positive control, completely inhibited GFP translation. Several of the selected peptides showed concentration-dependent effects on GFP translation. Figure 3 shows the results for DIRTQRE, TYLPWPA, TLWDLIP, CVRPFAL, and FVRPFAL peptides at 1 mM. From these *in vitro* protein translation inhibition assays, CVRPFAL and FVRPFAL showed the best inhibitory effects (compared to DIRTQRE, GFP fluorescence for those peptides was four-fold lower at 120 min). They both showed a similar level of inhibition, which could be due to the presence of a common motif, -VRPFAL.

The order of translation inhibition by peptides (from high to low) is as follows: CVRPFAL ≈ FVRPFAL > TLWDLIP > TYLPWPA > DIRTQRE. Concentration-dependent inhibitory effects of the peptides were observed for all peptides (Supplementary Material, Figure S1). Although this experiment does not reveal the target sites of peptides or the mechanism of translation inhibition, it does demonstrate that the selected peptides have the ability to inhibit translation by binding to either the translation or transcription machinery. Therefore, the next step was to determine whether the selected peptides bind to bacterial ribosomes. The peptides that demonstrate favorable binding to either ribosomes or free 16S rRNA could be used as further leads for refinement by peptidomimetic-based approaches.

### 2.4. In vitro binding to bacterial 30S ribosomes

**Figure 4:**
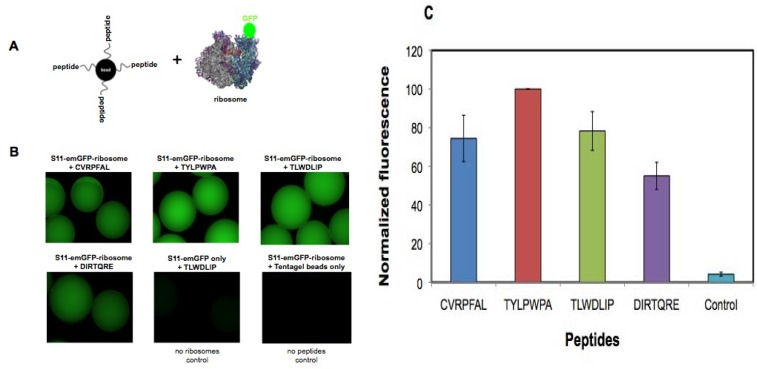
A) *In vitro* binding of selected peptides to isolated ribosomes. The peptides were synthesized on Tentagel beads and incubated overnight at 4 °C with GFP-S11-labeled 30S subunits in binding buffer (20 mM HEPES, pH 7.3, 10 mM MgCl_2_, 100 mM KCl). After washing with the same buffer, the peptides on beads with bound 30S subunits were visualized under a fluorescence microscope. Experiments with individual peptides were carried out in duplicate. B) Tentagel–peptide beads following ribosome binding are shown. Fluorescence is observed for peptides CVRPFAL, TYLPWPA, TLWDLIP, and DIRTQRE. The controls in which TLWDLIP beads were incubated with S11-emGFP protein (no ribosomes) or Tentagel beads only (no peptide) were incubated with S11-emGFP-ribosomes and did not show any fluorescence. C) The fluorescence levels on the beads were quantified and normalized, taking the fluorescence value observed by the TYLPWPA peptide as 100%. The other peptides were compared with TYLPWPA. Data were determined from two beads for each peptide and three points on each bead. The average was taken from two separate experiments.

The purpose of this experiment was to determine whether the selected peptides have affinity for bacterial ribosomes. The binding affinities of various peptides could also be compared by this method. Small ribosomal subunits, 30S, labeled with GFP on ribosomal protein S11 were added to peptides covalently attached to Tentagel beads (Figure 4A). GFP-tagged S11 protein (His(6)-EmGFP-S11) was over-expressed in *E. coli* DH5 and the resulting fluorescently labeled ribosomes were isolated by sucrose gradient centrifugation. The peptides were chemically synthesized on Tentagel beads with their N-termini exposed. Increasing concentrations of GFP-tagged 30S subunits were added to the Tentagel-bound peptides and unbound 30S subunits were removed by washing. Relative fluorescence intensity of the 30S subunits bound to the peptides on bead was determined by imaging on a fluorescence microscope, with the assumption that the level of fluorescence on the beads was proportional to the affinity of the ribosome for the peptide sequence. 

Peptides selected against modified h31 were shown to have affinity for 30S subunits (Figure 4B). The fluorescence intensity of each set of beads containing the selected peptides was quantified. Of the four peptides tested (CVRPFAL, TYLPWPA, TLWDLIP, and DIRTQRE), TYLPWPA showed the highest relative binding affinity to 30S subunits and DIRTQRE showed the lowest relative affinity (Figure 4B and C). As a control, purified His(6)-EmGFP-S11 protein was added to the TLWDLIP peptide on beads to determine if the fluorescence obtained was due to non-specific binding of protein to beads. In this case, fluorescence was not observed, suggesting that the fluorescence observed with the ribosome samples was due to interactions between the 30S subunit and the peptide. In the absence of peptides, the beads themselves did not show any binding to His(6)-EmGFP-S11. 

These results demonstrate that a component of the 30S ribosome has affinity for the selected peptides. The order of binding preference by the 30S subunits (from high to low) is as follows: TYLPWPA > CVRPFAL ≈ TLWDLIP > DIRTQRE. These results are not entirely consistent with the coupled *in vitro* transcription-translation assay; however, both of these assays involve many components (RNA, proteins, etc.) that may influence the binding affinity or activity of the peptides, which were selected to bind to only a small component of the rRNA. Nonetheless, the results are promising in that positive responses with respect to both binding and activity are observed for the selected peptides.

### 2.5. In vitro h31 binding studies

**Figure 5:**
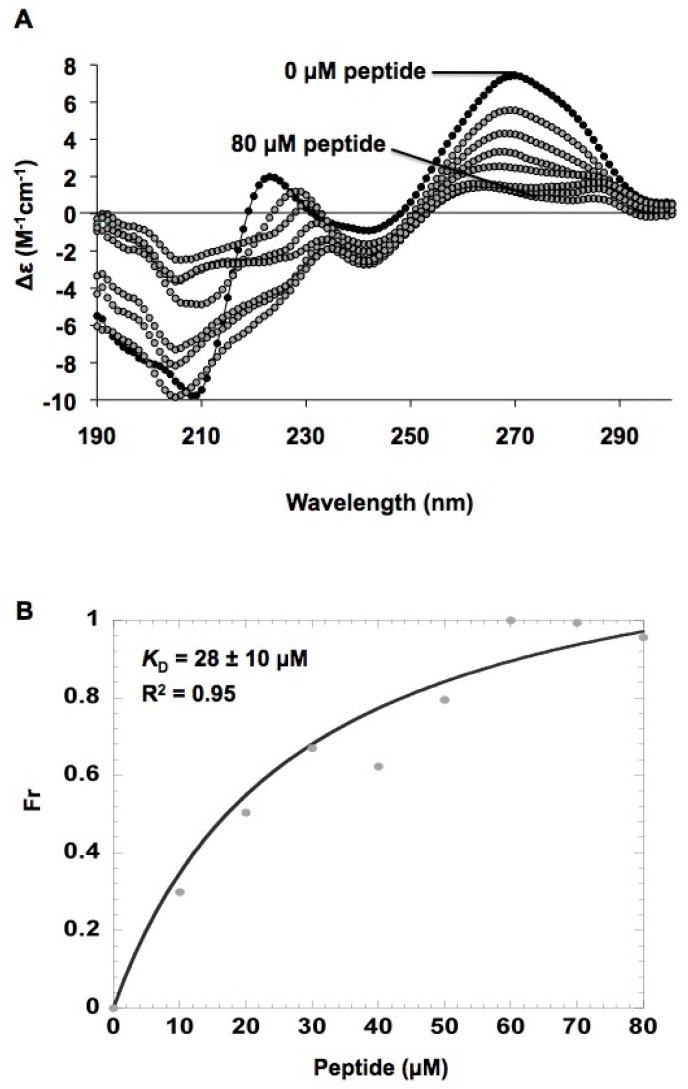
A) Circular dichroism (CD) changes in modified h31 upon the titration of peptide TYLPWPA. The buffer contained 100 mM NaCl, 0.1 mM Na_2_EDTA, and 10 mM sodium phosphate at pH 7. Each spectrum is an average of three scans. Black circles; CD spectrum from 190 to 300 nm of the wild-type h31 RNA prior to the addition of peptide; grey circles; spectra with increasing peptide concentration (0–80 µM). The spectra are shown as molar ellipticity (Δε, M^-1^cm^-1^). B) Curve fitting for determination of an apparent *K_D_* is given. RNA-peptide binding was determined by reading the Δε changes at a constant wavelength of 270 nm upon addition of peptide to the sample. The Δε value at 270 nm for each spectrum was converted to a fraction bound ratio (Fr) and the dissociation constant (*K_D_*) of RNA-peptide binding was determined by curve fitting using the Kaleidagraph™ 3.0 program.

Circular dichroism (CD) was employed to assess the conformational changes in h31 upon binding of the peptide TYLPWPA. A sample containing 0.5 μM wild-type, modified h31 (without biotin) was titrated with increasing concentrations (0–80 μM) of peptide (100 mM NaCl, 10 mM sodium phosphate, 0.1 mM Na_2_EDTA, pH 7.0) to obtain the spectra shown in Figure 5A. Conformational changes in h31 upon binding of TYLPWPA were observed. The decrease in ellipticity for RNA upon increasing peptide concentration implied a decrease in helicity of the hairpin RNA. Such findings may reflect a destabilization of the hairpin RNA upon peptide binding. The relative changes in molar ellipticity at 270 nm were determined, and a plot of fraction bound versus peptide concentration with a hyperbolic curve fit (Figure 5B) gave an apparent dissociation constant, *K_D_*, of 28 ± 10 μM (R^2^ = 0.95) for the TYLPWPA-RNA interaction. In general, the CD data gave poor fits, possibly due to cooperative binding or peptide aggregation effects. Thus, other methods were necessary in order to verify the binding interaction between the selected peptides and h31 RNA.

**Figure 6:**
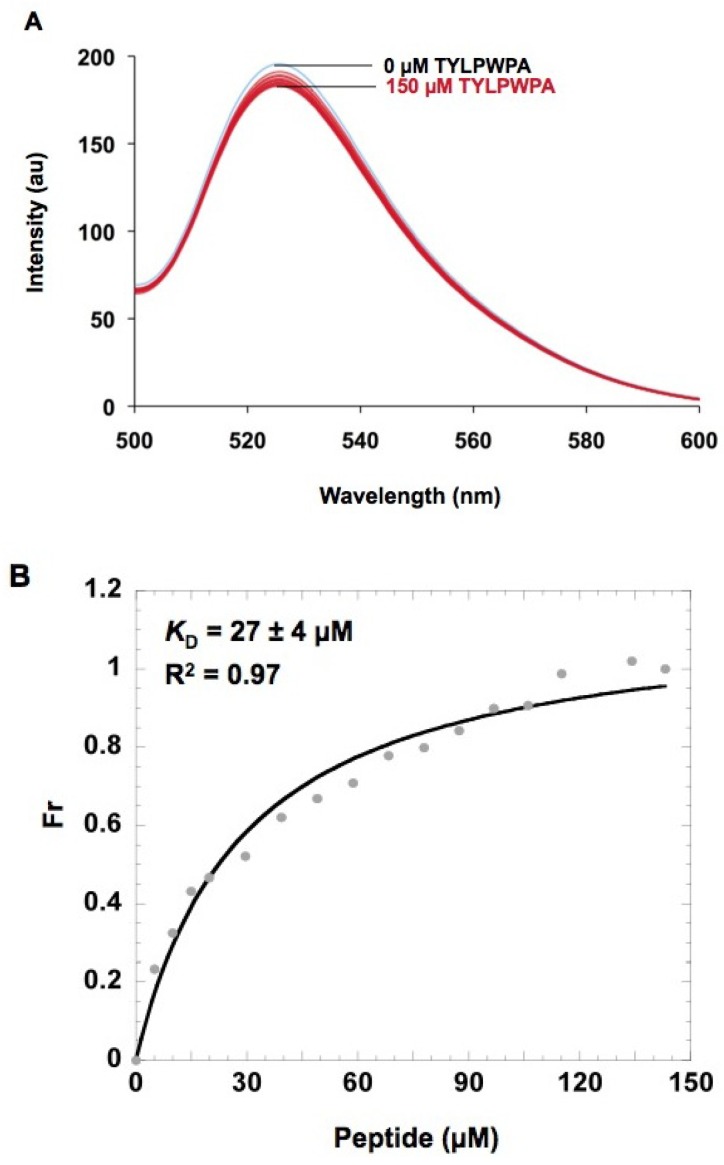
A) Fluorescence spectral changes associated with TYLPWPA binding to F-h31 (5'-fluorescein-labeled, wild-type h31). The buffer contained 50 mM NaCl, 1 mM Na_2_EDTA, and 10 mM HEPES at pH 7.5. The emission spectra were recorded in the range from 500 to 600 nm (excitation wavelength of 490 nm). B)Determination of the apparent *K_D_* for the fluorescence titration of peptide TYLPWPA with h31. The fluorescence intensity at 523 nm was converted to a fraction bound ratio (Fr) and the dissociation constant (*K_D_*) of RNA-peptide binding was determined by curve fitting using the Kaleidagraph™ 3.0 program.

Modified h31 was 5′ labeled with fluorescein (referred to as F-h31) in order to monitor possible conformational changes in h31 upon peptide binding [62]. A sample containing 0.5 μM of F-h31 was titrated with increasing concentrations of TYLPWPA (0–150 μM) (10 mM HEPES, 50 mM NaCl, 1 mM Na_2_EDTA, pH 7.5). In this experiment, the fluorescence intensity decreased upon increases in the peptide concentration (Figure 6A); however, the percent change in the observed fluorescence intensity was fairly small (7%). One possible reason for this observation is quenching of the fluorescein fluorescence by the adjacent 5′-guanosine residue [63,64]. A plot of the fraction bound versus peptide concentration and hyperbolic curve fitting gave an apparent *K_D_* of 27 ± 4 μM (R^2^ = 0.97) (Figure 6B*)*. A Hill plot analysis indicated a single binding site (n = 1) for modified h31; however, the relatively poor curve fits reflect the fact that the fluorescence changes are quite small. Even though the fluorescence and CD titrations gave similar *K_D_* values for the TYLPWPA peptide (*K_D_* = 27–28 µM), other methods were necessary to verify the binding affinities of the peptides to h31 RNA. 

Modified h31 was labeled with biotin in order to immobilize the RNA onto a surface and determine the kinetics of peptide binding by surface plasmon resonance (SPR). Initial experiments performed with the peptide TYLPWPA as the analyte gave negligible responses, likely due to the relatively small size of the peptide compared to the RNA. In order to enhance the signal-to-noise ratio, a GFP-protein-fused peptide, TYLPWPA-GFP, was generated and used as the analyte. 

Due to its larger size, the peptide fused with GFP has a greater change in refractive index on the SPR surface, hence generating a significant signal response upon binding to RNA. Biotinylated, modified h31 RNA was immobilized onto a streptavidin-coated CM5 chip and differing concentrations of TYLPWPA-GFP (10–100 µM) were injected as the analyte. A range of salt concentrations, detergent levels, pH conditions, and flow rates were tested, since these factors affect the peptide-RNA complex formation (data not shown). The apparent dissociation constants were determined under the following optimized conditions: 10 mM Tris·HCl, 150 mM NaCl, 10 mM MgCl_2_, 1 mM dithiothreitol (DTT), 0.005% (v/v) P20 at pH 7.5 as the running buffer at a flow rate of 50 μL/min.

**Figure 7:**
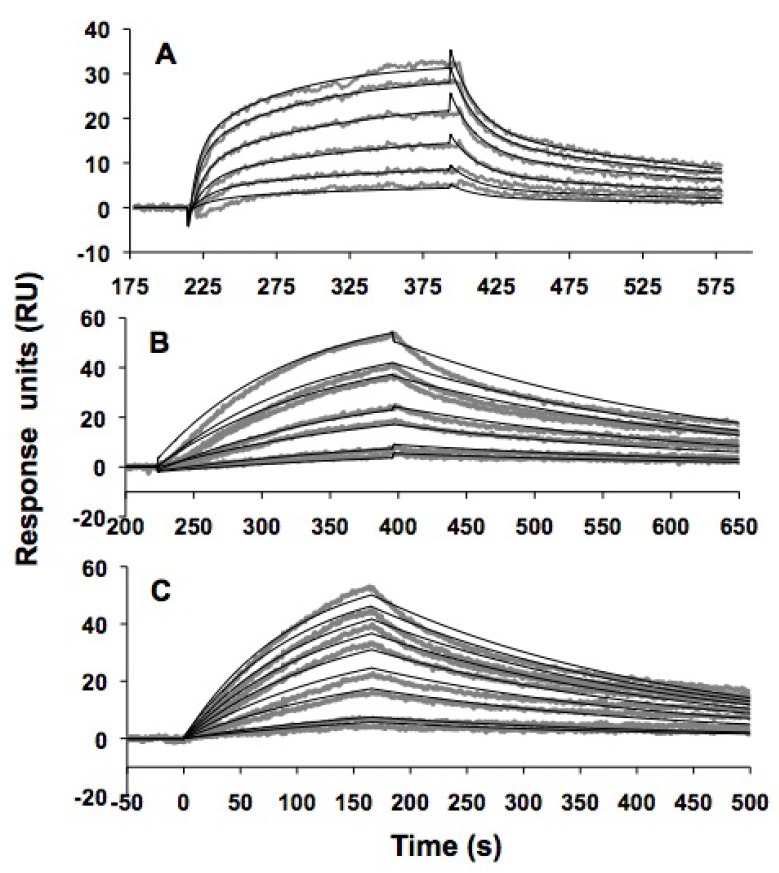
Surface plasmon resonance (SPR) binding curves and curve fittings obtained from the kinetic analysis of wild-type h31 binding to (A) TYLPWPA-GFP (10–100 µM), (B) TLWDLIP-GFP (0–1 µM), (C) CVRPFAL-GFP (0–1 µM). The 5'-biotinylated wild-type h31 RNA (~50 fmol) was immobilized onto the SPR sensor surface (CM5) and titrated with the peptides fused to GFP at varying concentrations.

The SPR results for TYLPWPA-GFP and modified h31 are shown in Figure 7A. Peptides TLWDLIP-GFP (0–1 μM) and CVRPFAL-GFP (0–1 μM) were tested for binding to modified h31 under identical conditions (Figure 7B and 7C, respectively). The sensorgrams generated were transformed and overlay plots were prepared using BiaEvaluation software. The data were fit to a Langmuir 1:1 binding model to obtain the kinetic data given in Table 4. 

**Table 4:** Kinetics and affinity data of a 1:1 interaction between wild-type modified h31 and GFP-tagged peptides obtained through SPR kinetic analysis^a^.

	TYLPWPA-GFP	TLWDLIP-GFP	CVRPFAL-GFP	GFP
***k_a_*** **(M^-1^s^-1^)**	(1.50 ± 0.02) × 10^2^	(93 ± 2) × 10^2^	(120 ± 1) × 10^2^	(2.22 ± 0.03) × 10^2^
***k_d_*** ** (s^-1^)**	(4.2 ± 0.04) × 10^-3^	(3.1 ± 0.3) × 10^-3^	(2.6 ± 0.02) × 10^-3^	(4.4 ± 0.04) × 10^-3^
***K_D_***	**28 ± 1 μM**	**330 ± 7 nM**	**230 ± 3 nM**	**20 ± 1 μM**
**χ^2^**	0.5	2.1	1.4	1.3

^a^The data were fit to a 1:1 binding Langmuir model using BIAevaluation 3.0 software. *k_a _*= association rate constant, *k_d _*= dissociation rate constant and the ratio between *k_d_* and *k_a_* gives the reported dissociation constants (*k_d_/k_a_=K_D_*).

The results with TYLPWPA-GFP were similar to those obtained with the free peptide and h31 or F-h31 by CD and fluorescence (*K_D_* of 28 µM), respectively. In contrast, TLWDLIP-GFP and CVRPFAL-GFP were determined by SPR to bind to modified h31 RNA with higher affinities (*K_D_*s of 330 and 230 nM, respectively). Surprisingly, free GFP was shown to have a *K_D_* of 20 µM for modified h31 RNA, which is comparable to TYLPWPA-GFP, but 60- to 80-fold higher than the peptides TLWDLIP-GFP and CVRPFAL-GFP. At this time we do not fully understand the basis for binding of GFP to the modified h31. SPR studies with unmodified h31 did not reveal any binding interactions with CVRPFAL-GFP (Supplementary Material, Figure S2). These results are very encouraging, since CVRPFAL was identified from the selection against modified h31 and counter selection with unmodified h31. 

The CVRPFAL peptide was chosen over FVRPFAL for these initial binding studies because it was obtained from the selection against modified h31 and counter selection with unmodified h31; whereas, the FVRPFAL peptide was only selected against modified h31. Nonetheless, FVRPFAL showed promising results in the *in vitro* translation assay and should be more chemically stable than CVRPFAL. Therefore, future work will include using this homologous peptide in binding studies with modified h31.

### 2.6. Discussion

The nucleoside modifications in h31 of small-subunit rRNAs are conserved among bacteria, but differ from the modifications in the corresponding helices in the *Archaea* and *Eukarya*. Using a heptamer phage-display library, two sets of peptides with different specificities for the bacterial h31 hairpin were identified. One set of peptides was selected against the unmodified h31 hairpin and the second set was selected against the fully modified hairpin containing m^2^G966 and m^5^C967. Peptides selected against wild-type h31 were shown to have affinity for both the synthetic modified h31 hairpin and 30S ribosomes isolated from *E. coli.* Our results demonstrate that h31-binding peptides CVRPFAL and TLWDLIP inhibit *in vitro* protein synthesis, which is consistent with recent studies demonstrating the importance of h31 in protein synthesis [28]. Although direct binding studies still have to be established, these results suggest that h31 is available for ligand binding *in situ* [57,65] and further establishes h31 as a potential new drug target. 

The fact that peptides CVRPFAL and TLWDLIP also have binding affinities (high nM) to modified h31 that are comparable to known antibiotics targeting 16S rRNA (e.g., paromomycin binding to the A site of 16S rRNA) [66,67] further indicates their potential to serve as drug leads. Further analysis to identify the key interactions responsible for specificity of the peptide for its target are currently underway. The on-bead, ribosome-binding assay will be applicable to screening one-bead one-compound libraries. With these data in hand, peptidomimetic and medicinal chemistry to optimize binding, solubility, membrane permeability, and bioavailability will be performed to enhance the feasibility of using these peptides for the treatment of bacterial infections. Furthermore, the presence of unique modifications in h31 [22,23] within a given domain may allow the use of similar approaches to identify leads for the treatment of diseases caused by non-bacterial pathogens such as fungi.

## 3. Materials and Methods

### 3.1. Media and bacterial strains

All plasmids were maintained and expressed in *E. coli* DH5 (*supE*44, *hsdR*17, *recA*1, *endA*1, *gyrA*96, *thi-*1, *relA*1) [68]. Recombinant M13 phage from the Ph.D.-7 heptapeptide library (New England Biolabs, Beverly, MA) were amplified and propagated in the *E. coli* host, ER2738 (F'*lacIq* *Δ(lacZ)*M15, *pro*A^+^B^+^*zzf*::*Tn*10(Tet^R^)/*fhu*A2, *supE*, *thi*Δ(*lac*-*pro*AB), Δ(*hsd*MS-*mcr*B)5, [r_k_^-^m_k_^-^McrBC^-^]), as described by the manufacturer. 

Bacterial cultures were maintained in LB medium [69] or LB medium containing 100 μg/mL ampicillin (LB-Ap100), LB medium containing 20 µg/mL tetracycline (LB-Tet), LB medium containing 35 µg/mL chloramphenicol (LB-Cm), or LB medium containing 30 µg/mL kanamycin (LB-Kan). Strains were transformed by electroporation [70] using a Gibco-BRL Cell Porator. Unless otherwise indicated, transformants were grown in SOC medium [68] for 1 h prior to plating on selective medium to allow expression of plasmid-derived genes.

To induce synthesis of peptide-GFP-His(6) fusion proteins for SPR experiments, *E. coli* EcpI300 cells were transformed with the fusion construct and grown in LB-Cm. L-Arabinose was added to a final concentration of 0.2% when the A_600_ = 0.1, and the culture was incubated for an additional 10 h. Fusion proteins were purified by immobilized metal affinity chromatography (IMAC) [71] using IMAC resin (Bio-Rad, Hercules, CA). 

To induce synthesis of His(6)-GFP-S11 used to create fluorescently labeled ribosomes, *E. coli* DH5 cells containing the His(6)-GFP-S11 construct were grown to A_600_ = 0.1 in LB-Kan medium, L-arabinose was added to a final concentration of 0.2%, and the cultures were incubated until A_600_ = 0.6, pelleted and stored at -20 °C prior to lysis in the French press and fractionation.

### 3.2. RNA sample preparation

The 18-mer RNA hairpins used in this study were chemically synthesized, purified, and characterized as previously described [54]. For circular dichroism experiments, h31 RNA was directly used without further processing or labeling. For the fluorescence experiments, the wild-type modified h31 construct was 5′ labeled with fluorescein. This procedure involves the use of a phosphorothioated h31 RNA construct, which was generated by reacting the RNA with ATP-γ-S and T4 polynucleotide kinase, followed by reaction with 5-iodoacetamidofluorescein (5-IAF) [72,73]. For phage selection and SPR experiments, the wild-type modified h31 construct was 5′ labeled with biotin. The biotinylated RNA can be immobilized onto a streptavidin-coated sensor chip or streptavidin-coated magnetic bead. The biotinylation reaction was performed with the phosphorothioated h31 RNA construct and *N*-iodoacetyl-*N*-biotinyl-hexylenediamine (NIBH). The biotinylated, unmodified h31 was generated by chemical synthesis at the Keck facility (Yale University, New Haven, CT). The labeled RNAs were purified by HPLC as described previously [54]. The purity of the labeled RNAs was then determined by 20% denaturing PAGE and by MALDI-TOF mass spectrometry. 

### 3.3. Biopanning of h31 target

The phage display method and phage titers were carried out as described by the protocol for the Ph.D.-7 heptapeptide library (New England Biolabs, Beverly, MA). The RNA targets were 5' biotinylated as described in the previous section. For each selection 25 µL of magnetic Dynabeads (M-280 streptavidin beads, Dynal Biotech, Oslo, Norway) were added to a PCR tube. The beads were washed three times with 100 µL of solution A (0.1 M NaOH, 0.05 M NaCl). During each wash step, the tubes were set on a magnetic holder while the supernatant was removed. Beads were then washed once with 100 µL of buffer B (0.1 M NaCl) and then once with binding buffer A (10 mM Tris·HCl, pH 8.0, 1 M NaCl). Fifty pmoles (or 30 pmoles in later rounds) of biotinylated modified h31 rRNA were added to the beads and incubated for 30 min at RT. At the same time, another set of beads washed with buffer A and buffer B were washed with RNA/phage washing buffer (10 mM Tris·HCl, pH 7.5, 10 mM MgCl_2_, 150 mM NaCl, 1 mM DTT). Ten µL of phage library (10^11^ phage particles), 8 µL of 5 µg/mL tRNA was added to the beads, washed with RNA/phage washing buffer and incubated for 30 min at RT. This step was done to prescreen the phage library against beads and tubes. Target-bound tubes were washed with RNA/phage binding buffer A, prescreened phage library was added to it and incubated at room temperature for 30 min. Tubes were washed with 200 µL of RNA/phage washing buffer containing 0.1% Tween-20. Bound phage were eluted by addition of glycine (0.2 M glycine, pH 2.2). The eluted phage pool was amplified in *E. coli* ER2738 and the procedure was repeated for subsequent rounds, but without prescreening against magnetic beads. The stringency of washing was increased by increasing the number of washes and amount of Tween-20 in consecutive rounds. At the end of the third and fourth rounds, eluted phage were amplified and PCR was performed on the plaques to amplify the region containing peptide clones and sequenced in-house with a Li-Cor 4200 (Lincoln, NE) global IR^2^ sequencer.

### 3.4. General procedure for the Fmoc heptapeptide synthesis

All syntheses utilized Rink amide resin (Novabiochem^®^, Gibbstown, NJ) preloaded with a 4-(2′,4′-dimethoxyphenyl-Fmoc-aminomethyl)-phenoxyacetamidonorleucylamino-methyl linker (Fmoc = 9-fluorenylmethoxycarbonyl). This resin has a theoretical substitution level of 0.68 mmol/g. Generally, 250 mg of resin was used for each synthesis. Therefore, 0.17 (0.68 × 0.25) mmol of theoretical sites were available for coupling. First, 250 mg of resin was loaded into the peptide synthesis reaction vessel mounted on a wrist-action shaker. The initial suspension and swelling of the resin (45 min) took place with shaking using dichloromethane (DCM) followed by washing with dimethyl formamide (DMF). The procedure consisted of iterative deprotection, coupling, and washing steps. Fmoc deprotection was accomplished by treatment with piperidine-DMF (25% (v/v); 10 × the resin volume) for 10 min with shaking, and a repeat with fresh deprotection solvent for 10 min, followed by DMF (5 times with 10 × the resin volume) and DCM (5 times with 10 × the resin volume) washing. Sequential coupling of residues involved mixing of Fmoc amino acid (3 × 0.68 × 0.25 mmol), DIC (*N,N*′-diisopropylcarbodiimide) (4 × 0.68 × 0.25 mmol), HOBt (1-hydroxy-1*H*-benzotriazole) (6 × 0.68 × 0.25 mmol), and DMF (5 × the resin volume) with gentle shaking for 2 h. The side-chain-protected Fmoc amino-acids were purchased from Novabiochem^®^. The Kaiser test was used to confirm complete coupling, as indicated by a negative result [74,75]. If the coupling was incomplete, additional DIC (4 × 0.68 × 0.25 mmol) was added and the vessel was shaken for an additional 2 h. If the coupling was complete, then the solution was drawn off, and the resin was washed with DMF (10 × the resin volume). 

Coupling and deprotection steps were repeated for each added residue, with intervening washing steps (DMF, 10 × the resin volume). After the final Fmoc deprotection, the resin was washed with DCM, DMF, ethyl ether, and acetone (twice each, 10 × the resin volume). Finally, resin cleavage solution [5 × the resin volume, TFA/TIS/thioanisole/anisole, 92:4:2:2 (v/v); TFA = trifluoroacetic acid, TIS = triisopropylsilane] was added with shaking for 2 h. The solution was collected and separated equally into two 50 mL centrifuge tubes, followed by addition of ethyl ether (-80 °C) to reach 80% of the total tube capacity. The solution was mixed, and the peptide precipitate formed immediately. After centrifugation (8 min at 6,000 rpm), the supernatant was decanted, fresh ether was added, and the pelleted peptide was mixed prior to another round of centrifugation (repeated three additional times). Finally, the peptide was dissolved in distilled water (5–10 mL), frozen, and lyophilized for 24 to 48 h until a white powder was obtained. 

### 3.5. HPLC purification of peptides

The crude peptides required HPLC purification prior to binding studies. This step eliminated salt as well as other contaminants, such as failed sequences, organic reagents, etc., which may otherwise interfere with the binding studies. Purification was performed on a Luna^® ^(Phenomenex, Torrance, CA) C18 reverse-phase column (250 × 10 mm) with water (0.05% TFA) as the mobile phase A and acetonitrile (0.05% TFA) as the mobile phase B at a flow rate of 5 μL/min. As a starting point, the lyophilized peptide was dissolved in water to form a 8–10 mg/mL solution, and a portion was tested in a gradient from 90% to 40% mobile phase A over a period of one hour. These data were used to optimize the procedure during subsequent runs. For bulk purification, lyophilized peptide was dissolved in water to give a 50–100 mg/mL solution, depending on the peptide solubility. The relevant fraction was collected and lyophilized to obtain the pure peptide. 

### 3.6. Quantification and analysis of peptides

The concentrations of peptides containing tryptophan or tyrosine were calculated based on the molar extinction coefficients (ε_280_) of these residues (ε^Trp^ = 5560 and ε^Tyr^ = 1200 AU/mmol/mL) [76]. The concentrations of GFP-fused peptides were determined by using a Micro BCA protein assay kit (Pierce™, Rockford, IL) [77]. The peptides were analyzed by MALDI-TOF mass spectrometry. The observed mass values are as follows: TLWDLIP [878.33 (M+Na^+^)], CVRPFAL [804.21 (M+H^+^)], FVRPFPL [874.26 (M+H^+^)], TYLPWPA [846.33 (M+H^+^)], and DIRTQRE [916.36 (M+H^+^)] 

### 3.7. In vitro transcription-translation inhibition assay

The inhibitory effects of selected peptides (DIRTQRE, CVRPFAL, FVRPFAL, TLWDLIP and TYLPWPA) on cellular processes were determined by *in vitro* coupled transcription-translation inhibition assay. The PURExpress™ *in vitro* protein synthesis kit #E6800S from New England Biolabs (Beverly, MA) was used for this purpose. A DNA template for GFP (pRSETEmGFP plasmid, Invitrogen) was employed at a concentration of 10 µg/mL in a 15 µL of reaction volume. Different concentrations of peptides were added, ranging from 300 µM to 2 mM. The DNA template was added only after mixing of the peptides or streptomycin so that they could bind to their target region first. For the control, streptomycin was added at a final concentration of 300 µM. The reaction was incubated in 384-well, Costar, clear-well, black plates and *in situ* incubated at 37 °C in a Gemini XPS microplate spectrofluorometer (Molecular Devices, Sunnyvale, CA) and the amount of GFP produced was monitored at different time intervals from 0 min to 2 h by fluorescence (excitation at 487 nm, emission at 509 nm).

### 3.8. Ribosome-binding assay

Four peptides isolated from the phage-display experiment, CVRPFAL, DIRTQRE, TLWDLIP, and CVRPFAL, were synthesized on Tentagel (Rapp Polymer, Tübingen, Germany) beads using standard Fmoc synthesis [78,79]. The beads were stored in DMF until use. The beads were then washed three times with 450 μl of water, and three times with 450 μL of the binding buffer B (binding buffer: 20 mM HEPES, pH 7.3, 10 mM MgCl_2_, 100 mM KCl), using 0.45 μm MWCO Nanosep (Pall Corporation, Ann Arbor, MI). The beads were then centrifuged at 6,000 rpm for 1–2 min. 

To prepare fluorescently labeled ribosomes, His(6)-EmGFP-S11 was cloned into the pACYC177 derivative, pKAN5T1T2 (Cunningham lab, to be described elsewhere). The resulting construct contains the His(6)-EmGFP-S11 fusion under transcriptional regulation of a P_BAD_ promoter [80,81]. Expression of the His(6)-EmGFP-S11 fusion in *E. coli* DH5 produces active, fluorescently labeled 30S and 70S ribosomes. Fluorescently labeled 30S subunits were isolated by sucrose gradient centrifugation in 5 mM Mg^2+^ [82]. For control experiments, His(6)-EmGFP-S11 was purified using IMAC resin (Bio-Rad, Hercules, CA) and the fluorescence was assayed using beads carrying peptides with ribosomes, beads carrying peptides with His(6)-EmGFP-S11 (no ribosomes) and beads only with ribosomes (no peptides). 

The fluorescent ribosomes were incubated in binding buffer B (20 mM HEPES, pH 7.3, 10 mM MgCl_2_, 100 mM KCl) at 37 °C for 30 min. Next, 25 pmoles of ribosome solution was added to each peptide, and the mixture was incubated overnight at 4 °C with agitation. Unbound ribosomes were removed by two 450 μL washes with binding buffer B and centrifugation at 6,000 rpm for 1–2 min. The beads were resuspended in 40 μL of binding buffer B for microscope screening and kept on ice until scanning. The beads (20 μL) were spread on microscope slides for scanning (Olympus 1X71 microscope, TH4 100 Olympus lamp, Olympus UCMAD 3 camera, 20× magnification)*.* An average of two pictures were taken per slide, eliminating beads presenting uneven swelling and cracks. 

### 3.9. Circular dichroism (CD) studies

All CD measurements were done on a Chirascan™ circular dichroism spectrophotometer at 25 °C using a 1 cm pathlength quartz CD cuvette. The modified h31 RNA was renatured by heating to 90 °C for 2 min followed by placing on ice. The titration was performed by adding increasing concentrations of peptide (10–80 μM) to 1 mL of 0.5 μM modified h31 RNA in 10 mM sodium phosphate, 100 mM NaCl, 0.1 mM Na_2_EDTA at pH 7, and incubating for 2 min for each concentration point followed by scanning from 190 to 300 nm (each spectrum is an average of three scans). RNA-peptide binding was determined by observing CD changes at 270 nm upon addition of peptide to the sample. The CD at 270 nm was converted to a fraction bound ratio and the dissociation constant (*K_D_*) of RNA-peptide binding was determined by curve fitting using the Kaleidagraph™ 3.0 program. 

The binding curve was determined by plotting Fr against the total peptide concentration, [P], in which Fr is the fraction of CD intensity (or ellipticity, θ) due to the bound species at a given wavelength. The observed CD intensity (ellipticity, θ) due to bound species at 270 nm was converted to a fraction bound ratio using Fr = (θ0,corr – θi,corr)/(θ0,corr – θf,corr), in which θ0,corr, θi,corr, and θf,corr are the corrected (to account for volume changes) CD values for RNA at the initial point (0, free), i^th^ point in the titration, and the sample at the final (f) point in the titration, respectively. The dissociation constant (*K*_D_) of RNA-peptide binding was determined by plotting Fr versus peptide concentration, and by curve fitting to Equation, Fr = [P]^n^/([P]^n^+*K_D_*^n^) where [P] is the peptide concentration, n is the Hill coefficient, and *K_D_* is the apparent dissociation constant (Figure 5B).

### 3.10. Fluorescence binding methods

Fluorescence was measured with a RF-5301PC spectrofluorometer (Shimadzu) at 25 °C using a 1 cm path-length quartz fluorometer cell. The emission spectra were acquired by scanning between 500 and 600 nm with an excitation wavelength of 494 nm, slit widths of 3 nm for excitation and emission. The fluorescent titrations were performed by adding increasing concentrations of peptide (5–150 μM) to 350 μL of 0.5 μM F-h31 RNA in HEPES buffer (10 mM HEPES, 50 mM NaCl, 1 mM Na_2_EDTA at pH 7.5) and incubated for 2 min for each concentration point followed by scanning. Prior to titration, the RNA was renatured. RNA-peptide binding was determined by quenching of the fluorescence intensity upon addition of peptide to the sample. The fluorescence intensity at 523 nm was converted to a fraction bound ratio and the dissociation constant (*K_D_*) of RNA-peptide binding was determined by curve fitting using the Kaleidagraph™ 3.0 program as described above for the CD titration. 

### 3.11. SPR methods

All immobilization and subsequent binding studies were performed using a BIAcore 2000 instrument set at a temperature of 25 °C. Standard dextran surface BIAcore sensorchips (Sensor chip CM5) were purchased from BIAcore (GE Healthcare, Piscataway, NJ). Standard desorb and sanitize routines were performed according to the BIAcore guidelines before docking a new CM5 sensor chip. All buffers were filtered through sterile 0.2 μm nylon membranes (Millipore™) under vacuum. Before derivatizing the flow cells with streptavidin, the CM5 sensor chip was pre-conditioned using three successive 10 μL injections of chip-preparation solution (10 mM NaOH, 500 mM NaCl) at 100 μL/min, followed by a normalization routine using BIAnormalizing solution (40% glycerol). Streptavidin derivatization was performed according to standard procedures [83,84]. 

Prior to immobilization, the solution of biotinylated RNA in HBS-EP buffer (10 mM HEPES, 3 mM EDTA, 150 mM NaCl, 0.005% P20 at pH 7.4) was renatured by heating to 90 °C for 2 min, cooling gradually to room temperature, and placing on ice. Flow cells were functionalized by injecting 30 μL of RNA at a time using the MANUAL INJECT command at a flow rate of 10 μL/min, until the desired immobilization level was reached. One of the flow cells was used to immobilize RNA, while the adjacent cell remained unmodified to serve as a blank-control for matrix effects. Levels of RNA captured were calculated by subtracting response units after injection from response units before injection for each flow cell. After RNA immobilization, both flow cells were blocked with 100 μL of 1 mg/mL biotin at 5 μL/min.

For the SPR experiments, the peptides were cloned into plasmid pBacEmGH (Cunningham Lab, to be described elsewhere) under transcriptional regulation of a P_BAD_ promoter. Upon induction with L-arabinose, the resulting construct produces a fusion protein in which the peptide is fused to the N-terminus of EmGFP containing a C-terminus 6 x His affinity tag (PEP-EmGFP-6 x His). 

Peptide samples were prepared by serial dilutions from stock solutions. Dilutions were made in running buffer (10 mM Tris·HCl, 150 mM NaCl, 10 mM MgCl_2_, 1 mM DTT, 0.005% (v/v) P20 at pH 7.5). All procedures for binding were automated, as optimized methods using repetitive cycles of sample injection and regeneration. Typically, running buffer was injected for ~30 min prior to experiment to establish a stable baseline value. Peptide solutions (200 μL) were injected at a kinetic flow rate of 50 μL/min, using the KINJECT command. All peptide samples were injected from 7 mm plastic vials (BIAcore) that were capped with pierceable plastic-crimp-caps to minimize carry-over and sample evaporation. Samples were injected in order of increasing concentration. The surface was regenerated using 150 μL of 500 mM NaCl. The data were fit to a 1:1 binding Langmuir model using BIAevaluation 3.0 software.

Kinetic constants were determined by integration of the experimental data using the differential rate equation *dRU/dt = k_a_·C·(RU_max_-RU)-k_d_·RU* to obtain *k_a_* and *k_d_* values simultaneously (*RU* = observed response, *RU_max _*= maximum response upon saturation, *C* = analyte concentration, *k_a _*= association rate constant, *k_d _*= dissociation rate constant). Then, the ratio between *k_d_* and *k_a_* gives the reported dissociation constants (*k_d_/k_a_=K_D_*). BIA evaluation uses Marquardt-Lavenberg algorithm to optimize parameters in fits and assigns kinetic constants to the above-described equation. The goodness of the fit was judged by the reduced chi-square (χ^2^) values.

## 4. Conclusions

The ultimate goal of this project was to identify ligands that bind bacterial h31 and can be used as drug leads. We identified peptide ligands that bind to wild-type h31 and unmodified h31 by M13 bacteriophage display. A cell-free protein translation assay was used to demonstrate that peptides bind to a component of the cellular machinery and inhibit protein translation. A ribosome-binding assay showed that these peptides have affinity towards 30S subunits. From surface plasmon resonance spectroscopy, we identified that CVRPFAL and TLWDLIP peptides are good ligands for h31 with *K_D_s* in the high nanomolar range (230–330 nM). This work indicates that these peptides are potential candidates for future drug leads. The results from this study will help to design novel peptides and peptidomimetics that can be used as antimicrobials in the future.

## Figures and Tables

**Figure A1:**
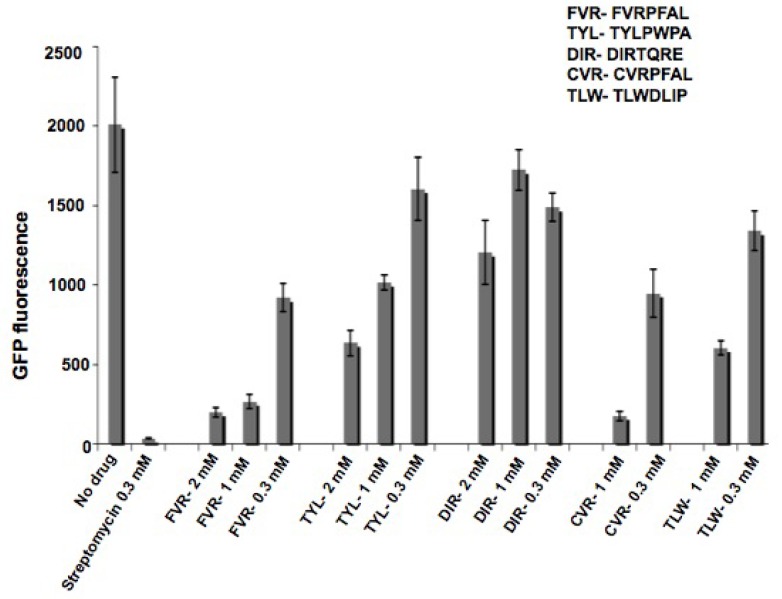
Concentration-dependent inhibitory effects of different peptides in a coupled cell-free transcription-translation system are shown.

**Figure A2:**
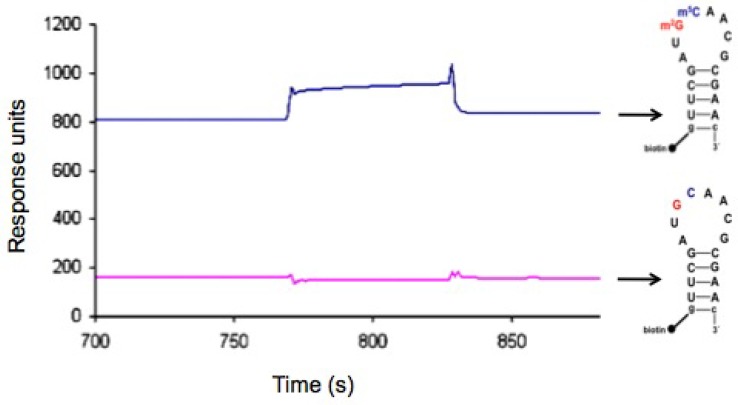
Comparison of the SPR binding data of CVRPFAL-GFP with wild-type h31 (upper sensorgram) and unmodified h31 (lower sensorgram). The amount of immobilized RNA was ~200 fmoles and the injection time was 1 min. The concentration of peptide was 1 μM. No binding interaction was detected with unmodified h31 under these conditions.

## Supplementary Materials

**Table S1:** Biopanning results for rounds 1–3 with wild-type modified h31.

Rounds	Input PFU	Output PFU	% yield
Pre-screen with beads	2.0 × 10^11^	2.5 × 10^10^	0.0125
I	2.8 × 10^11^	2.2 × 10^6^	0.08 × 10^-4^
II	2.5 × 10^11^	4.3 × 10^6^	0.17 × 10^-4^
III	2.2 × 10^11^	3.1 × 10^6^	0.14 × 10^-4^

**Table S2:** Peptides selected against streptavidin-coated beads^a^.

Peptides						
T	L	Q	P	G	G	A
S	L	L	A	H	P	H
H	L	E	N	H	P	M
S	L	V	S	H	P	M
H	L	E	N	H	P	M
G	L	D	H	H	P	P
I	P	E	W	H	P	Q
S	L	L	S	H	P	Q
T	L	L	A	H	P	Q
N	L	V	S	H	P	Q
I	P	E	W	H	P	Q
H	L	A	N	H	P	Q
S	L	L	A	H	P	Q
T	L	I	A	H	P	Q
S	L	I	A	H	P	Q
N	L	V	N	H	P	Q
N	L	L	N	H	P	Q
S	L	I	A	H	P	Q
I	S	S	T	H	P	Q
T	L	L	A	H	P	Q
I	P	E	W	H	P	Q
N	L	V	N	H	P	Q
T	L	L	A	H	P	Q
T	L	L	N	H	P	Q
T	L	L	A	H	P	Q
N	L	L	N	H	P	Q
T	L	I	S	H	P	Q
H	F	T	N	H	P	Q
I	A	P	N	H	P	Q
I	P	E	W	H	P	Q
T	L	L	N	H	P	Q
S	L	L	A	H	P	Q
T	L	I	S	H	P	Q
S	L	L	A	H	P	Q
N	L	L	N	H	P	Q
S	L	L	A	H	P	Q
I	P	E	W	H	P	Q
N	L	L	N	H	P	Q
N	L	L	N	H	P	Q
P	L	L	A	H	P	Q
S	L	L	A	H	P	Q
N	L	L	N	H	P	Q
S	L	L	A	H	P	Q
T	L	L	A	H	P	Q
I	P	E	W	H	P	Q
T	L	I	S	H	P	Q
T	L	I	S	H	P	Q
N	L	V	N	H	P	Q
S	L	I	A	H	P	Q
N	L	L	N	H	P	Q
G	Y	D	K	H	P	Q
S	L	I	A	H	P	Q
N	L	L	N	H	P	Q
I	P	E	W	H	P	Q
I	P	Y	W	H	P	Q
H	L	I	A	H	P	Q
S	L	L	S	H	P	Q
N	L	L	N	H	P	Q
T	L	L	A	H	P	Q
S	L	L	A	H	P	Q
N	L	V	N	H	P	Q
Y	L	V	N	H	P	Q
N	L	I	S	H	P	Q
N	L	L	N	H	P	Q
H	Y	E	G	H	P	Q
I	P	Y	W	H	P	Q
T	L	I	S	H	P	Q
I	P	Y	W	H	P	Q
T	L	L	A	H	P	Q
T	L	L	A	H	P	Q
I	A	P	N	H	P	Q
H	L	Y	A	H	P	Q
H	F	T	N	H	P	Q
N	L	L	N	H	P	Q
I	P	E	W	H	P	Q
H	L	Y	A	H	P	Q
N	P	T	K	H	Q	M
T	P	S	P	L	A	G
V	T	P	T	M	H	P
A	P	R	D	P	L	S
M	Q	S	H	Q	D	S
S	P	T	Y	Q	R	L
Y	M	E	H	S	R	V
H	R	I	S	W	P	S
D	P	F	F	Y	T	P

^a^One hundred phage isolates were sequenced after the fourth round of selection against streptavidin-coated beads. The known sequence motif for streptavidin is highlighted.

**Table S3:** Peptides selected against unmodified h31^a^.

Peptides												Frequency	
H	H	H		P		P		L		A		5	
H	H	P		P		F		P		P		2	
H	P	P		S		W		G		D		2	
H	P	P		H		F		P		N		1	
H	P	T		G		L		F		R		2	
H	N	H		L		G		V		H		2	
K	P	F		H		N		S		T		3	
K	P	F		H		N		S		T		2	
K	P	G		Y		S		S		A		2	
K	P	P		Q		V		P		L		2	
K	P	H		A		P		H		R		1	
K	P	P		H		H		P		R		3	
K	P	P		H		P		V		Y		1	
K	P	V		K		V		P		R		2	

^a^Two hundred phage isolates were sequenced after the fourth round of selection against unmodified h31 on streptavidin-coated beads. Only peptides with repeated sequences or common motifs are shown.

**Table S4:** Sequence homology of selected peptides with *E. coli* proteins.

Sequences	Homology with	Position
**TYLPWPA**	ATP dependent RNA helicase, Dead/DeaH box family	2 YLPWA 7 42 YLPWA 47
**FVRPFPL**	Glycine tRNA synthetase, beta subunit	1 FVRP 4 157 FVRP 160
**TLWDLIP**	23S rRNA m**5**U747 methyl transferase	2 LWDL 5 237 LWDL 240
	Leucyl-tRNA synthetase	1 TLW 3 771 TLW 773
**CVRPFAL**	Glycine tRNA synthetase, beta subunit	4 PFAL 7 477 PFAL 480
**DIRTQRE**	Predicted methyltransferase	4 TQRE 7 224 TQRE 227
	Valyl tRNA synthetase	2 IRTQR 7 63 IR - QR 67
**ATPLWLK**	Phenylalanine tRNA synthetase	2 TPLWLK 7 230 TPLWLK 235
**HHHPPLA**	Fused glutamine amidotransferase	3 HPPLA 7 384 HPPLA 388
**KPFHNST**	16S rRNA m**2**G1207 methylase	2 PFHN 5 270 PFHD 273
